# Supplementary material for: Virulence, Antibiotic Resistance, and Phylogenetic Relationships of Aeromonas spp. Carried by Migratory Birds in China
Source: Microorganisms. 2022 Dec 20;11(1):7. doi: 10.3390/microorganisms11010007 (PMC9862355; doi:10.3390/microorganisms11010007)
Supplement: Supplementary file 1 [file microorganisms-11-00007-s001.zip › microorganisms-2027234-supplementary.pdf]

Supplementary Material

Phylogenetic characteristics, virulence and antibiotic resistant of *Aeromonas* spp. carried by migratory birds in China

Bing Liang [1,2†*], Xue Ji [1,2†], Bo-wen Jiang [1,2], Tingyu Yuan [3], Chao Lu Men Gerile [4], Lingwei Zhu [1,2], Tiecheng Wang [1,2], Yuanguo Li [1,2], Jun Liu [1,2], Xuejun Guo [1,2] and Yang Sun [1,2,3*]

[1] Changchun Veterinary Research Institute, Chinese Academy of Agricultural Sciences, Changchun, Jilin, China
[2] Key Laboratory of Jilin Province for Zoonosis Prevention and Control, Changchun, Jilin, China
[3] Ruminant Diseases Research Center, College of Life Sciences, Shandong Normal University, Jinan, China.
[4] Center for Animal Disease Control and Prevention of Yi Jin Huo Luo Banner, Ordos, China
* Correspondence: bing897933468@163.com (B.L.); sunyang10@hotmail.com (Y.S.)

1 Supplementary Figures and Tables

1.1 Supplementary Figures

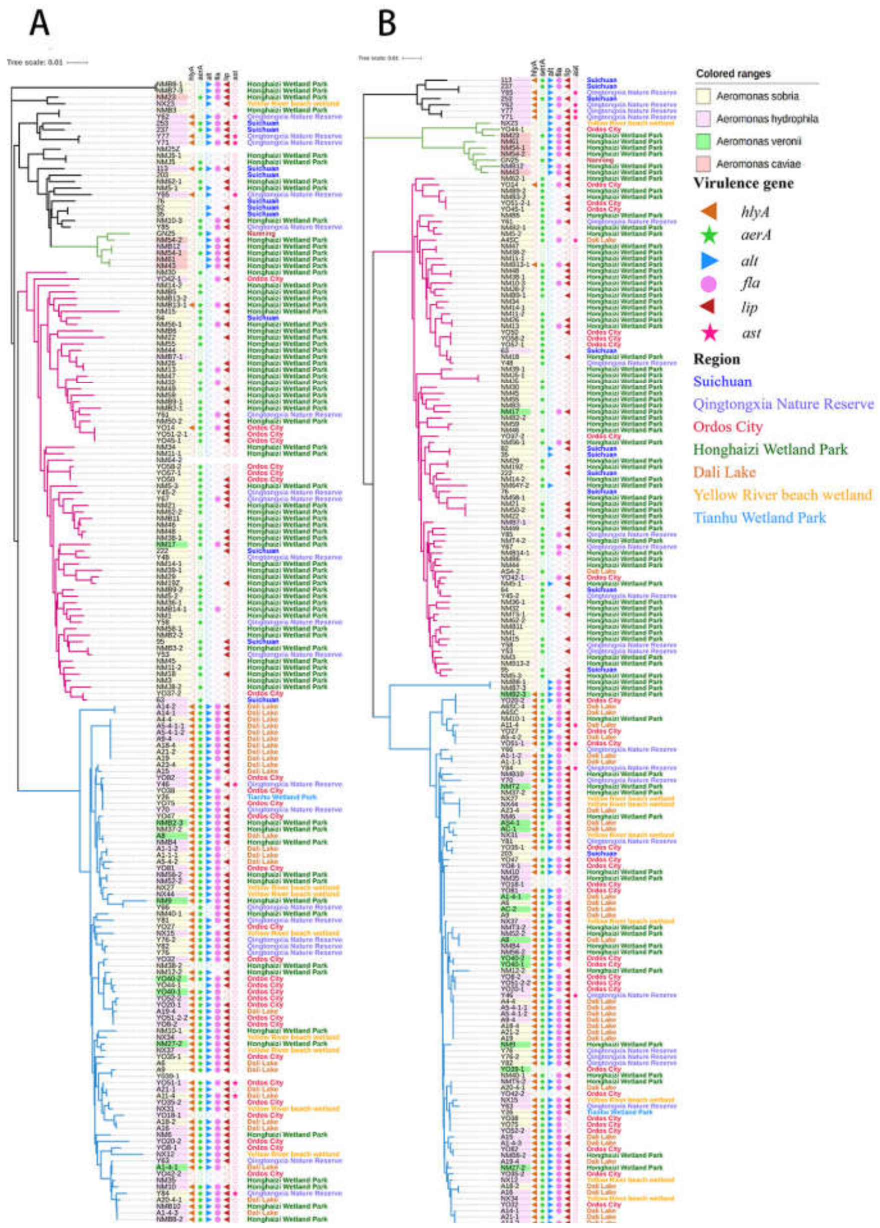

Supplementary Figure S1. Unrooted phylogenetic trees based on *recA* (A) and *metG* (B) gene sequences, showing relationships in the genus *Aeromonas* from migratory birds in this study. Note: A solid symbol shows that the strain contains virulence-associated genes; a hollow symbol shows that the strain did not contain virulence-associated genes. Colored rectangles represent the species of *Aeromonas*. For each strain, the shapes of different colors indicate the presence of the virulence factor genes analyzed in this study. The different color characters represent *Aeromonas* strains isolated from seven different regions in China.

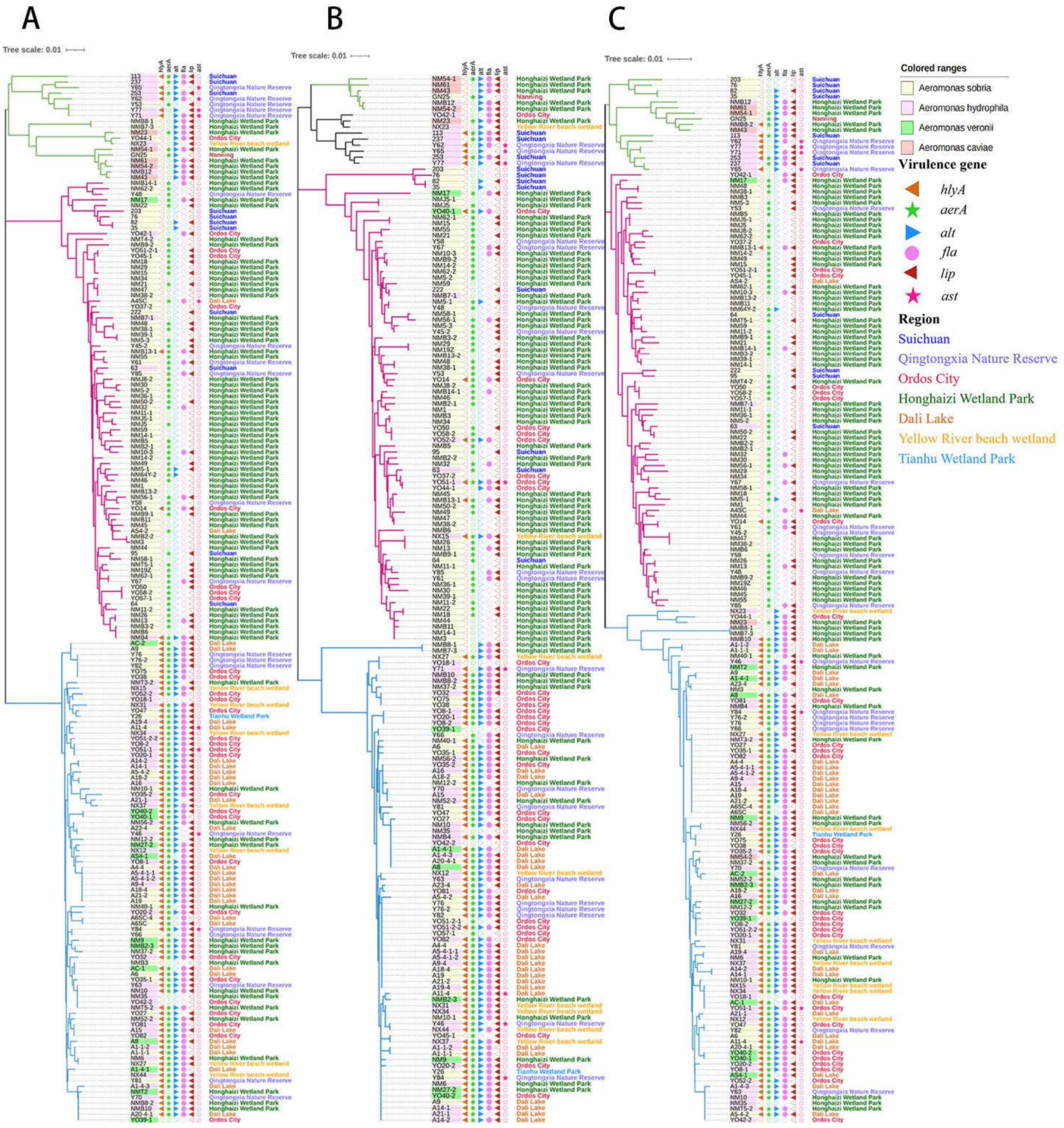

Supplementary Figure S2. Unrooted phylogenetic trees based on *groL* (A), *gltA* (B) and *gyrB* (C) gene sequences, showing relationships in the genus *Aeromonas* from migratory birds in this study. Note: A solid symbol shows that the strain contains virulence-associated genes; a hollow symbol shows that the strain did not contain virulence-associated genes. Colored rectangles represent the species of *Aeromonas*. For each strain, the shapes of different colors indicate the presence of the virulence factor genes analyzed in this study. The different color characters represent *Aeromonas* strains isolated from seven different regions in China.

1.2 Supplementary Table 1

Supplementary Table S1. 21 kinds of antibiotics in BD Phoenix TM-100 automatic microbial identification system

Antimicrobial classification	Antimicrobial agent
Aminoglycosides	Amikacin
	Gentamicin
Carbapenems	Imipenem
	Meropenem
First and second generation cephalosporins	Cefazolin
Third and fourth generation cephalosporins	Ceftazidime
	Cefotaxime
	Cefepime
Monocyclic lactams	Aztreonam
Penicillins	Ampicillin
	Piperacillin
Penicillins/ β- Lactamase inhibitor complex	Amoxicillin-Clavulanate
	Ampicillin-Sulbactam
Pseudomonas Penicillins- β- Lactamase inhibitor complex	Piperacillin-Tazobactam
Polymyxin	Colistin
Sulfonamides	Trimethoprim-Sulfamethoxazole
Chloramphenicol	Chloramphenicol
Quinolones	Ciprofloxacin
	Levofloxacin
	Moxifloxacin
Tetracyclines	Tetracycline

Supplementary Table S2. In vitro susceptibility of 176 *Aeromonas* isolates to 13 antimicrobial agents

Antimicrobial agent	Total（176）	Ningxia (29)	Jiangxi (11)	Honghaizi Wetland Park (80)	Ordos City (30)	Dali Lake (25)	Nanning (1)
Gentamicin	8.0%(14)	31.0%(9)	9.1%(1)	1.3%(1)	6.7%(2)	4.0%(1)	—
Cefazolin[a]	39.2%(69)	69.0%(20)	36.4%(4)	21.3%(17)	33.3%(0)	68.0%(17)	100.0%(1)
Ceftazidime	0.6%(1)	—	9.1%(1)	—	—	—	—
Cefotaxime	8.0%(14)	48.3%(14)	—	—	—	—	—
Cefepime	2.3%(4)	13.8%(4)	—	—	—	—	—
Aztreonam	2.3%(4)	13.8%(4)	—	—	—	—	—
Ampicillin	97.7%(172)	100.0%(29)	100.0%(11)	98.8%(79)	93.3%(28)	96.0%(24)	100.0%(1)
Piperacillin	13.6%(24)	58.6%(17)	27.3%(3)	2.5%(2)	6.7%(2)	0.0%()	—
Amoxicillin-Clavulanate	2.8%(5)	—	—	—	3.3%(1)	16.0%(4)	—
Ampicillin-Sulbactam	89.8%(158)	89.7%(26)	100.0%(11)	95.0%(76)	76.7%(23)	84.0%(21)	100.0%(1)
Piperacillin-Tazobactam	2.8%(5)	—	18.2%(2)	2.5%(2)	3.3%(1)	—	—
Colistin[b]	13.1%(23)	6.95%(2)	27.3%(3)	6.3%(5)	16.7%(5)	32.0%(8)	—
Trimethoprim-Sulfamethoxazole	11.4%(20)	58.6%(17)	—	2.5%(2)	3.3%(1)	—	—
Chloramphenicol	10.2%(18)	58.6%(17)	9.1%(1)	—	—	—	—
Ciprofloxacin	4.0%(7)	20.7%(6)	—	—	3.3%(1)	—	—
Tetracycline	14.8%(26)	58.6%(17)	—	2.5%(2)	20.0%(6)	—	100.0%(1)

[a] Refer to MIC standards for *Vibrio*

[b] Refer to EUCAST standards for *Enterobacter*.

Moxifloxacin was not analyzed statistically separately.

Note: Univariate ANOVA revealed that the difference between groups was highly significant for each antibiotic. Other pairwise comparisons within each group were significant. It was observed that isolates were susceptible to amikacin, imipenem, meropenem, and levofloxacin. Reference standards were not used for drug resistance of Aeromonas to mupirocin.

Supplementary Table S3. Antibiotic susceptibility test (AST) typing of *Aeromonas* spp.

The geographical position	AST profile	Phonotypic typing profile	Number of strain	MAR index
the north(53)	001000000000	C	7	0.08
	001001010000	D	9	0.25
	101101001111	G	5	0.67
	000000000000	H	3	0
	000001000001	I	3	0.17
	001001001101	K	4	0.42
	001101001101	L	2	0.50
	001111001101	M	2	0.58
	100001000000	N	2	0.17
	100001010000	O	2	0.25
	000000000001	P	1	0.08
	000000010000	Q	1	0.08
	000000011000	R	1	0.17
	000001011000	S	1	0.25
	001001000010	T	1	0.25
	001001001000	U	1	0.25
	001001110000	W	1	0.33
	001101000000	X	1	0.25
	001110001101	Y	1	0.50
	001110011101	Z	1	0.58
	100000000000	AA	1	0.08
	101001010000	AB	1	0.33
	101100001111	AC	1	0.58
	101101001101	AD	1	0.58
The south(2)	001001010100	V	1	0.33
	101101110000	AE	1	0.50
the north(111)&The south(10)	000001000000	A	79&5	0.08
	001001000000	B	22&2	0.17
	000001010000	E	4&1	0.17
	001001000001	F	4&1	0.25
	000001100000	J	2&1	0.17

Note: The order of AST profile is Aminoglycosides, Carbapenems, First and second generation cephalosporins, Third and fourth generation cephalosporins, Monocyclic lactams, Penicillins, Penicillins/ β - Lactamase inhibitor complex, Pseudomonas Penicillins- β - Lactamase inhibitor complex, Polymyxin, Sulfonamides, Chloramphenicol, Quinolones and Tetracyclines. 0: sensitive; 1: drug resistant

Supplementary Table S4. Genetic detection of six virulence genes in *Aeromonas* spp.

Target gene	Total (176)	Statistics by Region								Statistics by *Aeromonas* species			
		Ningxia (29)	Nanning (1)	Suichuan (11)	Honghaizi Wetland Park (80)	Ordos City (30)	Dali Lake (25)	The south (12)	The north (164)	*A.hydrophila* (53)	*A. sobria* (109)	*A. veronii* (9)	*A. caviae* (5)
hlyA	46.0%(81)	69.0%(20)	—	18.2%(2)	22.5%(18)	63.3%(19)	93.8%(37)	16.7%(2)	48.2%(79)	83.0%(44)	27.5%(30)	77.8%(7)	—
*aer*A	76.1%(134)	82.8%(24)	100.0%(1)	54.5%(6)	73.8%(59)	80.0%(24)	100%(83)	58.3%(7)	77.4%(127)	79.2%(42)	75.2%(82)	88.9%(8)	40.0%(2)
alt	52.3%(92)	72.4%(21)	100.0%(1)	45.5%(5)	32.5%(26)	63.3%(19)	78.1%(45)	50.0%(6)	52.4%(86)	88.7%(47)	31.2%(34)	77.8%(7)	100.0%(5)
fla	54.0%(95)	72.4%(21)	—	27.3%(3)	37.5%(30)	66.7%(20)	90.6%(50)	25.0%(3)	56.1%(92)	81.1%(43)	35.8%(39)	88.9%(8)	100.0%(5)
lip	64.2%(113)	86.2%(25)	—	54.5%(6)	56.3%(45)	60%(18)	81.3%(63)	50.0%(6)	65.2%(107)	79.2%(42)	55.0%(60)	66.7%(6)	100.0%(5)
ast	4.5%(8)	20.7%(6)	—	—	—	3.3%(1)	6.3%(1)	—	4.9%(8)	11.3%(6)	1.8%(2)	—	—

Note: Univariate ANOVA revealed that the difference between groups was highly significant for each antibiotic. Other pairwise comparisons within each group were significant.
